# Editorial: Perivascular niche and stem cell signaling in tooth

**DOI:** 10.3389/fcell.2025.1555739

**Published:** 2025-01-31

**Authors:** Takehito Ouchi, Emi Shimizu, Bo Li

**Affiliations:** ^1^ Department of Physiology, Tokyo Dental College, Tokyo, Japan; ^2^ Department of Oral Biology, Rutgers School of Dental Medicine, Newark, NJ, United States; ^3^ State Key Laboratory of Oral Diseases, National Center for Stomatology, National Clinical Research Center for Oral Diseases, Department of Orthodontics, West China Hospital of Stomatology, Sichuan University, Chengdu, Sichuan, China

**Keywords:** stem cells, dental, regeneration, mesenchymal stem/stromal cells, perivascular niche, tooth

Tooth is a unique mineralized hard tissue surrounding dental pulp tissue with trigeminal ganglion neurons and blood vessels. In addition, the tooth is supported by periodontal tissues composed of hard alveolar bone, cementum, soft gingiva, and periodontal ligament tissues. The function of the tooth is, thus, precisely and complicatedly achieved by these cells’ combination and healthy tissue integrity. Perivascular niche is the micro space that serves as a stem cell reservoir and is an essential part of maintaining physiological conditions and influencing the pathological conditions of dental tissues (Shi and Gronthos, 2003). Senescence is one of the most important elements in regulating stem cell ability (López-Otín et al., 2023). Most recently, Li et al. presented the first spatially resolved transcriptomic landscape of murine jawbone including dental tissues, and uncovered DNA methylation as a crucial mechanism underlying transforming growth factor beta 1 (TGF-β1)-induced periodontal ligament stem cell (PDLSC) senescence (Li et al., 2024). Ju et al. reported that ectonucleotide pyrophosphatase/phosphodiesterase (ENPP) 1 is crucial for TGF-β1-induced PDLSC differentiation. ENPP modulates the mineralization efficacy driven by adenosine triphosphate released from dental pulp cells in response to intrapulpal pressure (Techatharatip et al., 2018). In observing stem cell behavior, cell differentiation, maturity, and spatiotemporal understanding between stem cells and blood vessels, mouse incisor is a good model for tracing cellular genetic changes. In dental pulp, there are the fundamental cell-to-cell communications that orchestrate tooth formation, angiogenic-odontogenic coupling, a distinct mechanism compared to the angiogenic-osteogenic coupling in bones (Matsubara et al., 2022).

Tooth formation is a complex process controlled by genetic information and environmental factors (Murashima-Suginami et al., 2021). Because root dentin formation is an important part of tooth formation, it is valuable to clarify the precise control mechanisms of root dentin formation, and more specifically, the differentiation process of pulp stem cells in the tooth root. Due to the process of dentin formation in the root is different from that in the crown, several unique factors must be involved in the formation of root dentin. Identifying these unique factors may help understand the process of adult root dentin formation and root regeneration. Cui et al. harvested tissues from the labial and lingual sides of mouse incisors and conducted microarray analysis. Gene ontology (GO) analysis of differentially expressed genes indicated the critical role of extracellular matrix in the discrepancy of dentin formation between root and crown, for which hemicentin-1 (*Hmcn1*) was selected as the target gene. In addition, single-cell RNA sequencing analysis showed the expression pattern of *Hmcn1* at different developmental stages in mouse molars. The spatiotemporal expression of HMCN1 in mouse incisors and molars was detected by immunohistochemical staining as well. This group also investigated the functions of HMCN1 in human dental pulp cells, including proliferation, differentiation, and migration. Uncovering expression patterns of the spatially complicated but precisely differential gene expression will strengthen tissue area-specific target treatment in the future.

Both human and rodent molars have multiple roots and are formed through similar developmental sequences. It is considered that the formation of the tooth root and its surrounding structures including PDL and alveolar bone is important for tooth eruption (Ono et al., 2016). Tooth eruption, a crucial part of tooth development and regeneration, involves alveolar bone anabolism and catabolism. Periodontium, which surrounds teeth, is derived from dental follicle stem cells (DFSCs). During tooth eruption, DFSCs inhibit osteoclast differentiation by releasing extracellular vesicles containing Annexin A1 (ANXA1) and its mediated pathway, thereby preventing premature tooth eruption. Elucidation of this mechanism is extremely important for the understanding and treatment of abnormal tooth eruption diseases and the tooth regeneration process (Sun et al.).

Teeth and periodontal tissues are able to regenerate due to the inherent autonomous ability of their constituent cells. However, once huge deformation occurs, it is difficult to reproduce the integrated nature of tissue. This situation calls for regenerative therapies using bioengineering tools that maximize the potential of stem cells (Yang et al., 2019). Zheng et al. reported that N-acetylcysteine (NAC) is a stable, safe, and highly bioavailable antioxidant that shows promising prospects in bone tissue engineering due to the ability to attenuate oxidative stress and enhance the osteogenic potential and immune regulatory function of cells. This group systematically introduced the antioxidant mechanism of NAC, analyzed the advancements in NAC-related research involving mesenchymal stem cells (MSCs), precursor cells, innate immune cells, and animal models, discussed its function, and placed particular emphasis on the innovative applications of NAC-modified tissue engineering biomaterials.

Perivascular cells as MSCs in human dental pulp and periodontal tissue express NOTCH3 and they are very similar and composed of identical subpopulations. In addition to perivascular MSCs, endothelial cells, Schwann cells, and fibroblasts, etc. Construct perivascular niches (Pagella et al., 2021). Understanding the mechanisms of homeostasis and maintenance of dental perivascular niches by the intra and extracellular signals is essential to promote the development of dental regenerative medicine. Dental niche cells in tooth formation participate in tooth development, which may shed light on designing next-generation tooth bioengineering strategies to achieve the eventual goal of *de novo* tooth regeneration (Hu et al., 2022). In this regard, further studies on the formation of dental vascular niches and the involvement of bioengineering technology will further support the acceleration of dental stem cell regenerative medicine.
